# Transactions of the Buffalo Obstetrical Society

**Published:** 1884-11

**Authors:** 


### Society Reports.

### Transactions of the Buffalo Obstetrical Society.

*Stated Meeting, September 23, 1884.*

The President, Dr. W. W. Potter, in the Chair.

Dr. C. R. Jewett read a paper on " Puerperal Eclampsia."

He gave the history of the malady chronologically and medically, and its pathology as at present understood. The doctrine of cerebral anæmia, in its etiological relations to puerperal eclampsia, was elaborated in a forcible and plausible manner. The relations of the various forms of Bright's diseases to puerperal eclampsia were treated of, and the bearing of the former upon renal insufficiency, a prime cause of eclampsia, quite fully dilated upon.

The differentiation of uremia and eclampsia received a full measure of consideration at the hands of the essayist, and the unsatisfactory nature of the results in this direction thus far attained were pointed out.

The post mortem appearance of the kidneys was described, and the usually pale condition of these organs referred to as indicative of renal insufficiency. The essay was replete with evidence of pathological research and statistical accuracy, dealing almost wholly with the etiology and pathology of the malady.

*Discussion*—Dr. Banta, in commenting on the paper, remarked that he stood in greater fear of post partum hemorrhage, and puerperal eclampsia, than any other complications of pregnancy or parturition. He believes it to be of the first importance to address the treatment to the prevention of eclampsia, when it can be done, by the administration of diuretics and active cathartics. He further believed it good practice to give full doses of morphia, with a view to suspend the morbid processes, after the convulsions set in, and thought this would generally control eclamptic fits, which appear first *after* delivery.

Dr. Van Peyma thought it advisable, no matter what view we may take of the etiology or pathology of the disease, to induce labor at once when convulsions appear, and thus rid the system of the chief embarrassment to the reception of other therapeutic measures.

Dr. Hartwig had seen two forms of convulsions, one with and another without the presence of albumen in the urine. He believed that the urine should always be examined where there was suspicion of approaching eclampsia, but did not regard the test-tube as an infallible indicator, either of the certain coming on of the malady, or of the degree of its severity. He does not give credence to the theory of cerebral anæmia as causing the convulsions, but believes there is, on the other hand, hyperæmia of the brain when they appear. He would, among other things, administer sub-cutaneous injections of pilocarpin, in the treatment of the disease, and related cases of its beneficial employment in his hands.

Dr. Howell said his personal experience with eclampsia had been *nil*, but he had seen Schroeder, in Berlin, successfully treat the disease with chloroform by inhalation, and chloral per rectum.

Dr. Stockton thought the early delivery of the woman was often demanded, not only for her own safety, but for that of the child as well; and believed that this important fact should not be lost sight of, in making up our judgment as to the therapeutic management of cases when the fœtus is viable.

Dr. Lothrop related cases which had fallen under his observation during twenty-eight years' practice, one of which had been fatal. He succinctly passed in review the various remedies which seemed to offer the best hope of successful employment, named the special conditions in which the several plans were indicated, and called attention to the usefulness of venesection in certain cases of plethora with high arterial tension. He also directed attention to the use of morphia, hypodermically administered, and accepted the experience of others as to its efficacy in this disease. The importance of urinalysis during the last months of pregnancy was urged upon the members as a safeguard to the patient and a source of safety to the attending accoucheur.

Dr. Frederick called attention to a report he had recently observed, upon the employment of Norwood's tincture of veratrum viride in the disease, and remarked that the chief point in connection with its use was that it should be carried to the point of producing emesis, in order to get the greatest benefit from the drug.

Dr. W. D. Greene was in favor, generally speaking, of the early induction of labor in these cases, and pointed out the fact that Nature herself usually made the attempt to rid the uterus of its contents, clearly indicating thereby the propriety of the practice. He furthermore remarked upon the ease and rapidity with which labor can, in most cases, be artificially induced.

The President remarked upon the interesting character of the subject chosen by the essayist, as indicated by the discussion. In regard to the doctrine of cerebral anæmia causing eclamptic fits, how is it, then, he asked, that remedies like the bromides, chloral, etc., which have such a strong tendency to diminish the cerebral blood-pressure, act with such potency many times in these cases? Formerly he had been of the opinion that the gravid uterus acted mechanically upon the renal emulgent veins in such a manner as to produce these convulsions; but, of late, he had been more inclined to regard the mechanical obstruction to the excretion of the urine, as a more tenable ground to occupy in regard to the etiology of eclampsia. This obstruction may be, in part, by pressure upon the ureters and in part by catarrhal or other obstruction of the uriniferous tubules. The ureters thus become dilated, distorted, or stretched out, or, possibly, blocked up by catarrhal exudate, so that the passage of urine is impeded; nor do they regain their function fully for some time after delivery. Whatever view may be taken of the pathology of this malady, he continued, it would appear to be good practice to seek to empty the uterus of its offending contents whenever it can be done without too much officiousness. He described two extreme cases where he had recently employed the artificial induction of labor with success. He also remarked upon the importance of preventive measures when the patients are seen in season; of the usefulness of morphia in large doses, hypodermically, as recommended by Dr. Clark, of Oswego; and warned against the indiscriminate employment of blood-letting. Finally, he counseled against the treatment of this malady by any fixed or pre-formulated plan, but, rather, to approach each case free to adopt such measures as seemed best suited, after a careful survey of all its surroundings, history and symptoms; for it would appear that now one method or set of remedies would seem best adapted, and again another plan, quite different in character, would offer the greatest probabilities of success.

Dr. Jewett, in closing the discussion, said that he had purposely avoided introducing anything on the subject of treatment in the paper, believing that the discussion would elaborate that branch of the subject quite fully enough. He had not been disappointed in his expectations. The etiology and pathology of the disease would probably be less understood, in view of the recent researches, and he had, therefore, confined himself exclusively to their consideration. In regard to treatment, he did not regard the induction of labor of value, unless it be done before the occurrence of convulsions; any palliative is quite as good. Morphia, chloroform, chloral, etc., only suspended, for the time being, the morbid processes, did not remove them. Thinks pilocarpin used in diseases of the kidneys logically wrong, consequently inadmissible in eclampsia, on account of the involvement of the kidneys.

